# Endoscopic *en-face* optical coherence tomography and fluorescence imaging using correlation-based probe tracking

**DOI:** 10.1364/BOE.444170

**Published:** 2022-01-14

**Authors:** Manuel J. Marques, Michael R. Hughes, Adrián F. Uceda, Grigory Gelikonov, Adrian Bradu, Adrian Podoleanu

**Affiliations:** 1 Applied Optics Group, Physics and Astronomy, Division of Natural Sciences, University of Kent, Canterbury CT2 7NH, United Kingdom; 2 Institute of Applied Physics RAS, Nizhny Novgorod, Russia; 3 Both authors contributed equally to this publication

## Abstract

Forward-viewing endoscopic optical coherence tomography (OCT) provides 3D imaging *in vivo*, and can be combined with widefield fluorescence imaging by use of a double-clad fiber. However, it is technically challenging to build a high-performance miniaturized 2D scanning system with a large field-of-view. In this paper we demonstrate how a 1D scanning probe, which produces cross-sectional OCT images (B-scans) and 1D fluorescence T-scans, can be transformed into a 2D scanning probe by manual scanning along the second axis. OCT volumes are assembled from the B-scans using speckle decorrelation measurements to estimate the out-of-plane motion along the manual scan direction. Motion within the plane of the B-scans is corrected using image registration by normalized cross correlation. *En-face* OCT slices and fluorescence images, corrected for probe motion in 3D, can be displayed in real-time during the scan. For a B-scan frame rate of 250 Hz, and an OCT lateral resolution of approximately 20μm , the approach can handle out-of-plane motion at speeds of up to 4 mm/s.

## 1. Introduction

Endoscopic optical coherence tomography (E-OCT) allows high-resolution imaging of tissue to a depth of 1-2 mm beneath the surface. E-OCT was first demonstrated [1] in the 1990s, a few years after the very first report of OCT on the eye [2]. Since then, E-OCT has been commercialized for use in coronary arteries, complementing intravascular ultrasound for interventional guidance [3]. Other potential applications of E-OCT include imaging the epithelium of internal organs, particularly for early diagnosis of cancerous lesions or for lateral margin identification during endoscopic surgery [3].

E-OCT probes can be broadly divided into ‘side-viewing’ and ‘forward-viewing’ designs. Side-viewing probes involve deflecting the beam exiting a fiber by 90 degrees, using a prism or mirror or by angle-cleaving the fiber. Scanning can be achieved by rotating the fiber using a motor at the proximal end, outside of the patient [1,4,5]. Alternatively, the deflecting element may be fixed onto a micro-motor at the distal end of the probe, in which case the fiber and lens assembly itself does not need to rotate [6–8]. To improve tolerance to probe bending, it is possible to implement the side-viewing probe as a common-path interferometer [9]. Different optical configurations in the distal end, such as those involving the use of a diffractive lens [5], can also help to mitigate spectral losses when large spectral bandwidths are required for increased axial resolution. 3D imaging is achieved by moving the probe axially, obtaining ‘tunnel-like’ images. The side-viewing probe can be incorporated into a capsule for esophageal imaging without an endoscope [10]. Side-viewing probes can also have very small diameters; needle probes with diameters as small as a few hundred microns having been demonstrated [11]. Using distal motors, they can also achieve high scan rates (up to 4000frames/s ), particularly when coupled with optical sources allowing MHz A-line rates, such as Fourier-domain Mode-Locked Lasers [8]. A comprehensive review of side-viewing probes is provided in Ref. [3].

Side-viewing probes are ideal for imaging tubular-shaped structures such as vasculature or parts of the gastro-intestinal tract. They are less well-suited to general endoscopic use where it may be desirable to have a forward-looking image, such as at branches of the airways, in organs such as the bladder or the stomach, or for image-guided surgery. For this reason, forward-viewing probes have also been developed, although not successfully commercialized. There are two common approaches to miniaturization of forward-viewing scanning probes. The first, and most common, is to scan the fiber itself laterally, usually behind a lens [12]. It is also possible to integrate a miniature fiber-based lens into the fiber itself [13]. The second is to incorporate a miniature beam-scanning element using a micro-electromechanical (MEMS) mirror surface [14], although the need to fold the beam makes this less suitable for narrow-diameter probes. In either case, the scanning can either be in one direction only, producing 2D cross-sectional OCT images, or in two directions, allowing 2D *en-face* images and volumes to be assembled.

The design of forward-viewing OCT endoscopic probes is challenging in several respects. The probe would normally be introduced to the body via the working channel of an endoscope, limiting both the maximum possible probe diameter (to around 3 mm) and the length of the rigid tip. It is technically difficult to build a fast 2D scanning element at low cost and in a sufficiently small package. The field-of-view tends to be small since, unlike side-viewing probes which can rotate a full $360^{\circ }$ , forward-viewing probes only scan over a small arc. For example, while recent state-of-the-art forward-scanning compact probe heads can acquire several volumes per second, typical image sizes are 0.8-3.5 mm [12,13,15–19]. To maintain an acceptable depth-of-field for endoscopic use, lateral resolution is typically 10-30 µm, resulting in a relatively small number of lateral resolution elements in the image.

In this paper we attempt to address some of these difficulties by introducing a new concept whereby scanning along only one axis is performed using a distal scanning mechanism (the fast scan), and the raster scanning (or scanning along the orthogonal axis) is completed by a manual bending mechanism located at the distal end of a flexible endoscope. This relaxes the requirements on the distal scanning mechanism (reducing it to a single axis scan), and means that OCT volumes can be much larger along the manual-scan (slow-scan) direction than those that could be produced by any feasible miniaturized distal scanning mechanism within the probe itself. This new approach comes with its own challenges, particularly the difficulty of assembling volumes during manual scanning without artefacts due to irregular motion along the manual-scan direction and unwanted axial motion of the probe (discussed in more detail below). In our previous work using a 1D scanning endoscope and a robotic actuator to perform the slow-scan [20], volumes were assembled simply by assuming constant speed in the slow-scan direction and no axial motion of the probe. When using a manually-scanned endoscope, a constant scanning speed or direction cannot be assumed, and so the instantaneous speed of the probe must be determined. The probe may also move axially or laterally within the plane of the B-scan, and this motion must be corrected for.

We propose here a solution involving a speckle correlation-based algorithm for determining motion in the direction out-of-plane of the B-scans, and demonstrate the feasibility of the approach in simulated imaging experiments. The scanning probe incorporates a double-clad fiber for simultaneous fluorescence imaging, and we show that we can also assemble 2D fluorescence images from a combination of mechanical and manual scanning, using the data from the OCT channel for registration. While dual fluorescence/OCT imaging has been demonstrated by numerous reports using side-viewing probes [21–24] there have been comparatively fewer forward-viewing dual-mode probes [25]. Using manual scanning on one axis, we are able to obtain fluorescence images several millimeters in length.

### 1.1 State of the art in volume assembly

The problem of assembling volumes from 2D B-scans has previously been studied in the context of manually-scanned 2D ultrasound probes, and algorithms based on speckle correlation have been suggested [26]. Conversely, most reports on the assembly of OCT images with some elements of manual scanning focus on building B-scans from A-scans. This is in contrast to the situation faced here, where we have one axis of mechanical scanning, producing high-quality B-scans, and we wish to assemble volumes and *en-face* images via manual scanning in the second, slow-scan, direction. Nevertheless, there are some clear similarities between the two tasks.

For assembly of B-scans from A-scans, in 2009 Ahmad *et al.* [27] demonstrated a simple algorithm in which the Pearson cross-correlation coefficient is calculated between a reference A-scan and each subsequent A-scan. Once the correlation drops below an experimentally determined threshold, the current A-scan is added to the B-scan and the process is repeated (with the current A-scan becoming the new reference). To reduce the impact of structural features, the A-scan had a moving average filter of several resolution elements in size applied, and this filtered A-scan was subtracted from the unfiltered A-scan. Liu *et al.* extended this work in 2012 [28] by incorporating a theoretical model of speckle to determine the probe displacement corresponding to a specific correlation value without experimental calibration. In 2015, Wang *et al.* [29] showed that improved performance could be achieved by taking multiple correlations between pairs of A-scans with different time separations.

While these A-scan assembly techniques were developed mainly for manually-scanned OCT probes, similar techniques have been applied to proximally-driven side-viewing endoscopes in order to correct non-uniform rotational distortions (NURDs). Of particular relevance to this work, Uribe-Patarroyo *et al.* [30] noted difficulties with patient motion, occasional under-sampling and areas of tissue with low signal which inevitably mean that image-based tracking cannot provide a perfect correction. Aboeui *et al.* [31] adopted a more complex algorithm involving dynamic time warping. Other approaches to correct NURDs in side-viewing endoscopes include tracking reflections from the endoscopic sheath [32] or fiducial markers [33]; these methods are not applicable to a forward-viewing probe or the work reported in this manuscript.

The task faced here of assembling volumes from B-scans, where the slow-scan direction is manually scanned, is also related to the task of assembling volumes in side-viewing endoscopic OCT, where a manual pull-back of the probe acts as the second axis of scanning. Variations in the speed of the pullback cause distortions in the resulting volume. Lee *et al.* [34] proposed an approach in which two B-scans are acquired at two axial positions of the probe. As the probe moves axially, at some point the rear B-scan images the same point on the tissue as the front B-scan, and the time difference between these two events allows for the axial speed of the probe to be determined. A similar method was applied to a forward looking galvanometer-based [35] bench-top system but would be difficult to implement in a compact forward-viewing probe. More recently, Nguyen *et al.* [36] proposed a method to correct both for NURDs and longitudinal speed variations in side-viewing OCT. The longitudinal correction involved analyzing the statistical variation of intensity in the *en-face* image within a sliding window to estimate the pullback speed.

As discussed below, the method we adopted is closely related to the cross-correlation technique previously used to assemble B-scans from A-scans with manual scanning [27], but with the difference that mechanically-scanned B-scans are assembled into volumes (and fluorescence T-scans are assembled into fluorescence images). The lower frame rate of B-scans compared with the line rate of A-scans necessitates some modifications to the approach to allow for difficulty in ensuring over-sampling during manual scanning along the slow axis. As demonstrated below, this allows volumes to be successfully built when the probe is manually scanned using an endoscope at speeds of up to 4 mm/s.

## 2. Materials and methods

**Fig. 1.**
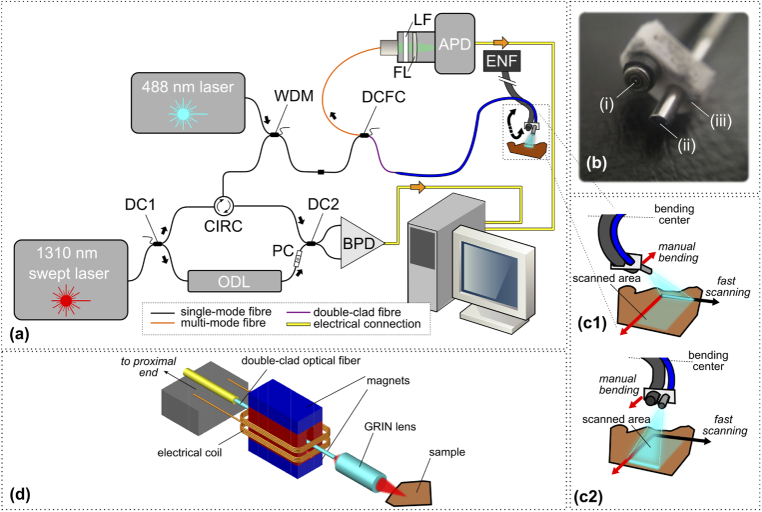
**(a)** Schematic diagram of the endoscopic SS-OCT/fluorescence system used in this study. **DC1-2**: fused fiber directional coupler; **CIRC**: optical fiber circulator; **WDM**: wavelength division multiplexer/combiner; **DCFC**: double-clad fiber directional coupler; **LF**: emission filter; **FL**: achromatic lens; **APD**: avalanche photo-detector; **ODL**: optical delay line; **BPD**: balanced photo-detector; **ENF**: Olympus ENF endoscope proximal controls. **(b)** Photograph of the Olympus ENF-P4 endoscope distal end (i), showing the OCT/fluorescence probe (ii) mounted to it using a 3-D printed bracket (iii). **(c1)-(c2)** Detail from (a), showing a close-up of the combined probe end with the Olympus endoscope at two manual scan positions. **(d)** schematic diagram showing the voice coil operated 1-D scanning probe.

A schematic diagram of the OCT/fluorescence endoscopic system used in this study is presented in Fig. 1. The system, loosely based on a configuration reported by Scolaro *et al.* [37], combines a swept-source OCT sub-system operating at a central wavelength $\lambda_{0}=1310\;nm$ with a fluorescence imaging sub-system, with a $\lambda_{e}=488\;nm$ solid-state laser providing the excitation. The two sub-systems share the same endoscopic probe (detailed in the next section), combined by means of a double-clad fiber coupler (DCFC, Thorlabs, model DC1300LEFA).

Briefly, in the OCT sub-system, the output from a MEMS swept-source (Axsun Technologies, central wavelength $\lambda_{0}=1310\;nm$ , tuning range Δλ=100nm , sweep rate 100kHz ) is sent to a fused fiber coupler DC1, with a splitting ratio of 90/10. 10% of the optical power is routed to a custom-built optical delay line (ODL) forming the reference arm of the interferometer, and is afterwards reunited with the power returning from the object arm at DC2, which has a 50/50 split ratio to ensure balanced detection at the photo-detector BPD (Thorlabs, model PDB480C-AC).

In the object arm of the OCT interferometer, the optical signal from the swept source is routed to a fiber-based wavelength division multiplexer (WDM, Font Canada, custom 488/1310nm fiber optic combiner) via a circulator, CIRC (AFW Technologies, model CIR-3-13-B-1-2-VR01), where it is combined with the output power from a 488nm solid-state laser providing the fluorescence excitation (JDSU, FCD488FC-020, 488nm wavelength, ≈20mW CW output power). Both optical signals are directed to the double-clad fiber coupler, DCFC (Thorlabs, model DC1300LEFA), where the combined OCT/fluorescence excitation power is routed to the double-clad fiber output, connected to the OCT/fluorescence probe, which is described in greater detail in Section 2.1.

The first cladding mode of the double-clad fiber collects any fluorescence signal from the sample, whereas the back-scattered OCT signal is routed through the core (fundamental) mode. In this way, the DCFC is capable of separating the returned OCT signal from the fluorescence signal. The latter is sent to an avalanche photo-diode amplifier module (APD, Hamamatsu model C5460-01, frequency response from DC to 100kHz ) via an emission filter LF (Thorlabs, model FBH520-40) and an achromat f=200mm lens FL (to ensure an optimum spot size on the active area of the APD), whereas the former is sent to the OCT interferometer via the WDM and CIRC. To provide fluorescence signal, the tissue in Section 4 was stained for approximately 2 minutes with acriflavine hydrochloride before rinsing with water.

Following detection of both OCT and fluorescence signals, their electrical representations are digitized into the computer (using, respectively, an AlazarTech model 9350 sampling at 500MS/s and a National Instruments model PCIe-6321, sampling at 200kS/s ). As mentioned in Section 1., we employ a custom-made software procedure to perform the volume reconstruction from the OCT B-scans acquired, which is described in detail in Sections 2.4-2.7. The reconstruction can occur offline (e.g. data is fully buffered and reconstructed afterwards), or concurrently with acquisition, in which case we refer to a real-time reconstruction, as discussed in Section 2.7.

### 2.1 OCT/fluorescence probe

The endoscopic probe is a forward-viewing, 1-D scanning probe, with an outer diameter of approximately 3 mm and a working distance of approximately 1 mm. Its working principle has been described elsewhere [20,38,39]; briefly, the probe operates on the voice coil principle, employing a cantilevered optical fiber which oscillates when driven by an alternating electric current (schematically shown in Fig. 1 (d)). The fiber tip is imaged onto the sample by means of a gradient-index (GRIN) lens (Edmund Optics, #64-545, 0.46mm working distance), creating a linear scan of up to 2mm in length, depending on the amplitude of the drive signal. Whereas the similar probe employed in our previous work [20] used a single-mode, SMF-28e fiber, in this case, due to the need to detect the fluorescence signal, we instead used a 1250-1600 nm double-clad fiber (DCF13, Thorlabs, Newton, NJ, USA, core diameter 9μm , first cladding diameter 105μm , second cladding diameter 125μm ).

### 2.2 OCT image reconstruction

In order to reconstruct OCT B-scans, the raw channeled spectra are processed using the Complex Master-Slave (CMS) method [40]. CMS replaces the Fourier transform usually employed in conventional OCT signal processing, presenting advantages such as tolerance to dispersion in the interferometer [41], long depth imaging due to the requirement for a swept-source *k*-clock being dropped [42], and improved reconstruction speed when only a small number of depth points are needed [43]. This last point is of potential relevance to this work, as the image reconstruction could be made to operate over a subset of the axial range, allowing *en-face* images to be assembled in real-time even for very high B-scan rates. However, this selective depth reconstruction was not necessary for the frame rates reported here. Further processing was also required to correct the distortion in the lateral direction due to the sinusoidal probe scanning drive signal. This was corrected by linear interpolation, assuming a sinusoidal scan, and was performed in real-time.

### 2.3 Endoscope integration

While the OCT probe is small enough to fit through a standard endoscope working channel, a suitable endoscope was not available when conducting this study. Instead, the OCT probe was fixed externally to an Olympus MODEL ENF-P4 endoscope, using a 3-D printed bracket as shown in Fig. 1(b)(iii). The Olympus endoscope has an outer diameter of 3.6mm and a bending range of up to 130 degrees, which allows the OCT/fluorescence probe to be scanned in the out-of-plane direction with respect to the B-scans. A similar approach of external fixing was previously employed for combined endoscopic and OCT imaging of the larynx [39]. In practice the OCT/fluorescence probe would normally be inserted through the working channel of a compatible endoscope, but otherwise the procedure would be similar.

When fixing the probe to the endoscope it was necessary to ensure that the direction of endoscope bending was approximately perpendicular to the OCT probe scanning direction. This was achieved by viewing the laser scanning line on an infra-red viewing card, rotating the OCT probe until the line was correctly oriented, and then fixing the probe in place.

### 2.4 Algorithm for assembly of volumes and *en-face* images

To be able to assemble volumes or *en-face* images from the raw B-scans while the probe is being manually scanned, it is necessary to estimate the instantaneous 3D velocity vector of the probe. Given that the probe is to be operated endoscopically, the exact path cannot be controlled or predicted (although the general direction of its movement can). Motion within the plane of the B-scans (axial or lateral) is straightforward to detect via image registration, while out of plane-motion is identified by looking for decorrelations in image speckle which cannot be explained by in-plane motion. Note that is out-of-plane motion which is required by the very process of scanning - and would be deliberately introduced by the operator - in order to acquire data to assemble volumes and *en-face* images. Both lateral and axial in-plane motion is undesirable but still likely to occur in practice.

A prototype of the algorithm for initial offline use was implemented in MATLAB (Mathworks). All code and a selection of example data is available in Code 1 [44]. A summary of the image processing algorithm is given below (and a graphical depiction of it is shown in Supplement 1, Fig. S3):

1. Beginning with the first B-scan ( i=1 ), the i th B-scan is selected as a reference image.
2. The average tissue surface height for the reference B-scan is identified. Several rapid methods for achieving this were investigated and are compared with manual segmentation in Supplement 1, Fig. S1. The method employed was binarization by thresholding of the image, followed by morphological opening and closing operations with a disk-shaped structuring element. The top surface position for each A-scan is then taken to be the location of the first non-zero pixel along the A-scan. The median of these positions across each A-scan is taken to be the average surface height for this B-scan.
3. For the (i+1) th (current) B-scan image, a region of interest (ROI) of width $\Delta x_{ROI}$ and height $\Delta y_{ROI}$ starting from a distance d above the median surface height is extracted. The 2D normalized cross-correlation (NCC) is computed between this ROI and the reference image.
4. The location of the NCC peak is identified, and this is taken as the lateral and axial shift between the two images. The current image is translated to correct for this shift.
5. To determine the out-of-plane motion, ROIs from the reference image and the current image are high-pass filtered by subtraction of the same image with a 2D mean filter applied, similar to the method proposed by Ahmad *et al.*[27]. This removes most structural content in the image, leaving behind the speckle pattern. A Gaussian filter is then applied to reduce noise. The ROI from the reference image is then thresholded to identify specularities and a binary mask created by applying a morphological open operation with a disc-shaped structuring element. A correlation is then performed between the pixels of the filtered ROI from the current and reference images which are not masked. This gives the degree of speckle decorrelation due to out-of-plane motion, with the mask preventing bright specularities from influencing the correlation calculation.
6. If the correlation is not below a predefined threshold, $c_{t}$ , indicating sufficient decorrelation for the *i*th image to be identified as an independent B-scan, Steps 2-5 are repeated for subsequent B-scan images, (*i* + 1), (*i* + 2) ⋯ , using the same *i*th reference image until the correlation value drops below the threshold.
7. If the cross-correlation peak value is below the threshold, this image is selected as the next B-scan to be used in the volume/*en-face* assembly. The image is laterally and axially shifted to correct for the shifts detected in Step 4 prior to insertion into the volume.

For the results presented below, the threshold for surface finding was set at twice the mean intensity value for the B-scan, and the disc structuring element employed had a diameter of 30 pixels ( 285×300 µm). The high-pass mean filter had a kernel size of 5×5 pixels ( 47.5×50 µm), the Gaussian filter had a sigma of 1.5×1.5 pixels ( 14.25×15 µm), the mask threshold was 5 times the mean pixel value in the ROI, and the size of the mask structuring element was 5 x 5 pixels ( 47.5×50 µm). The ROI was 180×100 pixels ( 1710×1000 µm), laterally centered and beginning immediately below the median detected surface height. Examples of intermediate processing steps for the surface finding are shown in Supplement 1, Fig. S2, and for the image processing prior to the correlation calculation in Supplement 1, Fig. S3. The effect of the location and size of the ROI is explored in Supplement 1, Fig. S4.

### 2.5 Handling under-sampling in the slow-scan direction by interpolation

The above method is expected to work when the manual scanning speed is relatively slow, such that there is over-sampling in the slow-scan direction. (This is similar to the situation that occurs when manually assembling A-scans in most previous reports [27]). However, when the probe moves more quickly, and the degree of over-sampling is reduced, the shift will be under-estimated. This is because, when a B-scan below the correlation threshold is obtained, no account is taken of how far below the threshold the correlation has fallen. Higher speeds are therefore under-estimated and the parts of the volume/*en-face* image acquired when the probe was moving faster will appear compressed in the out-of-plane direction. A modification to the algorithm was therefore developed to calculate the location of each B-scan relative to the previously inserted B-scan based on the actual value of the correlation of the first frame to drop below the threshold. Assuming an approximately linearly fall-off in cross-correlation peak with out-of-plane distance (see Section 3.2 for analysis of this approximation), the estimated distance moved, Δz , is given by

(1) $$\Delta z=z_{dec}\left(1+\frac{c_{t}-c_{m}}{1-c_{t}}\right)$$

where $z_{dec}$ is the experimentally determined out-of-plane movement distance required for the B-scan correlation to drop below the decorrelation threshold, $c_{t}$ , and $c_{m}$ is the measured correlation peak for this B-scan. Rather than simply inserting the B-scan as the next frame of the volume, the volume is then assembled by linear interpolation between the B-scans onto an evenly spaced grid in the out-of-plane direction. The interpolation points can be chosen to be equal to the lateral pixel size, resulting in images with square pixels, and hence the correct aspect ratio, without need for further scaling. The benefits of this approach are illustrated in Section 3.2.

### 2.6 Surface flattening

One of the aims of this study was to demonstrate that *en-face* images can be generated in real-time by manual scanning along the slow axis. *En-face* images are generally more useful if they follow the shape of the tissue surface rather than being an oblique slice through the tissue. As described above, axial shifts between B-scans are already corrected by the algorithm. While this was designed primarily to correct for probe axial shifts during acquisition, it will also tend to smooth out undulations in the tissue surface in the slow-scan direction, since these two effects are not easily separated. In addition to this, we also explored the possibility of correcting for a constant probe tilt along the fast scan axis, since again, if this is not corrected, this tilt would result in the *en-face* image being an oblique slice through the tissue. This is done by correcting for the average tilt of the tissue surface across all the B-scans of the volume. A volume was first assembled without lateral shift corrections. The volume was then re-sliced to obtain a stack of B-scans oriented along the slow scan direction. The median surface heights along each of these re-sliced B-scans (i.e. for each position in the fast scan) are then determined using the same method as described above. A linear fit to these surface heights gives an estimate of the probe tilt in the fast-scan direction. This measured tilt is then used to provide a simple correction for probe tilt (by axially shifting each A-scan of each B-scan) as part of a full reconstruction which includes both axial and lateral shifts.

### 2.7 Real-time implementation

To demonstrate the feasibility of assembling *en-face* images in real-time during endoscopic investigations, a simplified form of the algorithm was implemented in LabVIEW (National Instruments). While the majority of the algorithm was implemented using standard LabVIEW functions (including the Vision Development Module), cross-correlation and image filtering prior to speckle correlation measurement was performed using the OpenCV4 library, accessed from LabVIEW via DLLs compiled from C++. The implementation functioned similarly to the method described above, including tissue flattening in the slow-scan direction and interpolation. Probe tilt correction (i.e. flattening in the fast-scanning lateral direction) was not performed, as this requires information from the entire volume scan and hence cannot be implemented in a real-time *en-face* preview. OCT B-scans can be streamed directly to the *en-face* assembly algorithm from the OCT acquisition and processing software or from a saved datafile. Images were successfully processed in real-time at B-scan frame rates of 250 Hz, and the *en-face* image extracted at a fixed depth relative to the estimated tissue surface was displayed with line-by-line update as the probe was scanned. A video of the real-time display is provided in Visualization 1 and the LabVIEW code is provided in Code 2 [45].

## 3. Quantitative characterization and validation

### 3.1 Optical coherence tomography probe and system

The procedure to quantify the sensitivity of the OCT channel was the same as in a previous study from Marques *et al.* [20]. An A-scan was obtained from a brushed metal target and the signal to noise ratio (SNR) of the A-scan peak was measured with respect to the average noise floor to be 39.8dB . The reference arm was then blocked and the optical power was measured at the BPD first from the brushed metal and then from a mirror. The ratio of these measured powers was 35.5dB , and this was used to correct the measured signal-to-noise ratio of the signal from the metal to obtain a sensitivity of 75.3dB . This is much lower than 85−92dB in our other MS systems previously reported in the literature. This may be due to lower efficiency in re-injecting light back into the DCF inside the custom-made probe. The width of the A-scan peak was measured to be 28μm , which effectively dictates the axial resolution of the system.

**Fig. 2.**
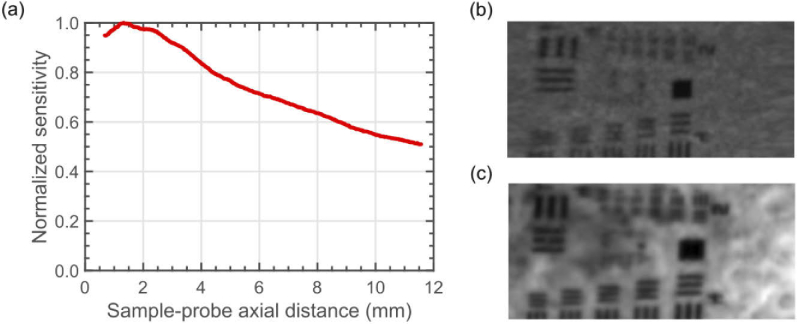
(a) Fluorescence signal as a function of distance from probe tip. A moving average filter of 5 points was applied to smooth the fluorescence sensitivity profile. (b) *En-face* OCT image of a positive USAF target. (c) Fluorescence image of positive USAF target placed over fluorescently-stained paper.

To measure the signal level of the fluorescence channel as function of distance from the probe, the probe was mechanically translated at constant speed away from a piece of paper stained with a 0.05g/100ml solution of acriflavine hydrochloride. The average signal across the fluorescence T-scan is plotted as a function of distance from the probe tip (determined from the position of the OCT-scan surface peak) in Fig. 2(a). Peak signal was obtained at 1.5 ± 0.1 mm from the probe, and there is no significant selection in depth for the fluorescence channel.

To characterize the lateral resolution of the probe, a positive United States Air Force (USAF) resolution target was placed on top of the stained paper. The probe was mechanically translated at constant speed across the sample at an optical distance of 2.5mm , without resorting to the B-scan-based reconstruction method. To this goal, a translation stage (Newport, model LTA-HS) was used. An *en-face* slice from the OCT channel and a fluorescence image are shown in Fig. 2(b) and (c), respectively. In the OCT channel, element 4 of group 4 can be resolved in both scanning directions, giving a lateral resolution of 22.1 µm. In the fluorescence channel, element 1 of group 5 can be resolved, giving a lateral resolution of 15.63 µm.

### 3.2 Characterization of volume/*en-face* assembly

To determine the relationship between speckle correlation and out-of-plane motion, the probe was translated over a scattering phantom (paper resolution target) and chicken breast tissue at 0.5 mm/s using the same motorized translation stage as in the previous section, perpendicular to the fast-scan direction (i.e in a direction that is out-of-plane of the OCT B-scan). The normalized cross correlation between the first image and a region of interest of 180×100 pixels ( 1710×1000 µm) extracted from each subsequent image was then calculated to determine in-plane shifts. The shift-corrected images were then processed as described above in Section 2.4, and the correlation as a function of out-of-plane motion distance was then calculated. This was performed 50 times, each with a different starting point within the scan, leading to a mean coefficient of variation of 14% across all data points, The correlation for zero shift was calculated by averaging the correlation between 20 pairs of images when the probe was not in motion to account for the effect of random noise.

**Fig. 3.**
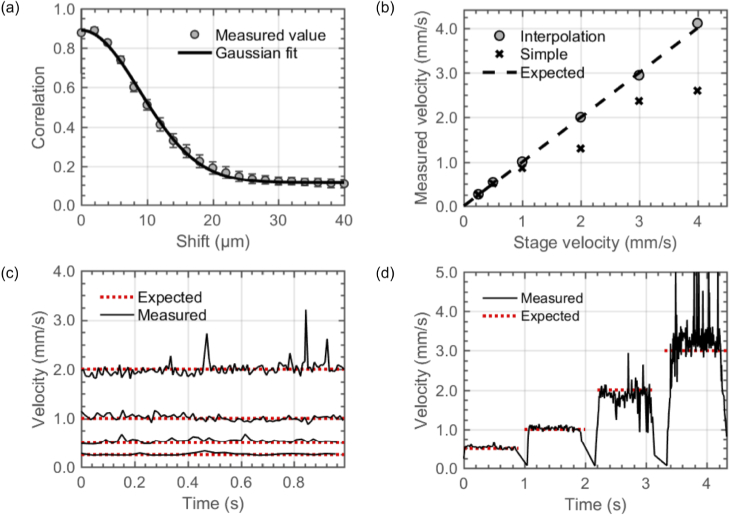
Validation of speed estimation in the out-of-plane direction via speckle decorrelation, using a mechanical translation stage to move the OCT probe over chicken breast tissue. (a) Correlation as a function of out-of-plane movement distance, average of 50 starting points. Error bars are standard deviation across 50 runs. A least-squares Gaussian fit is shown. (b) Measured speed using simple and interpolation based methods for six different velocities. (c) Speed measured as a function of time using interpolation method for four different translation stage speeds. (d) Speed measured as a function of time using interpolation method for probe motion with varying speed. Drops to zero speed are a mechanical feature of the way in which the stage was programmed, and are not artifacts of the method.

The mean correlation is shown as a function of out-of-plane distance for the chicken breast tissue in Fig. 3(a). The correlation drops to 0.5 at a distance of 10.4 µm. The curve is approximately linear between correlations of 0.7 and 0.3, which supports the use of the linear interpolation method described in Section 2.5. For the paper target, the correlation drops to 0.5 at a distance of 10.8 µm, suggesting that this calibration is not highly dependent on the sample properties, and so this data is not shown.

A Gaussian fit to the data of the form

(2) $$f(x)=(f_{max}-f_{min})\exp(-x^{2}/2\sigma^{2})+f_{min}$$

is also shown. σ , $f_{max}$ and $f_{min}$ are free parameters, found to be 12.0 µm, 0.952 and 0.052 for paper and 12.5 µm, 0.891 and 0.114 for the tissue, respectively. $f_{max}$ is the correlation between images when there is no out-of-plane motion, $f_{min}$ is the residual correlation at large distances, and σ is an estimate of the speckle grain size and hence the lateral resolution. Using the conventional definition of resolution as the full-width half-maximum (FWHM) of the point-spread function, this corresponds to an expected lateral resolution of approximately 21 µm in the out-of-plane direction, compared to the experimentally determined value of 22 µm in the in-plane direction.

This curve can be used to determine an appropriate value for the correlation threshold, $c_{t}$ . Clearly the value must lie in the range between $f_{min}$ and 1 or it will never be reached. As the threshold approaches $f_{min}$ the separation between B-scans will become reasonably large compared to the lateral resolution, while a threshold close to 1 will be more subject to random noise or other variations unrelated to out-of-plane motion. To follow the convention of sampling approximately twice per resolution element, a threshold of $c_{t}=0.5$ was adopted for the following work. The out-of-plane movement required to reach this decorrelation threshold is then $z_{dec}=10.4$ µm.

The speed of the probe in the out-of-plane direction can be estimated by determining how many B-scan frames are required for the correlation to drop below the threshold ( n ). The measured average speed, $\bar{v}$ , is then given by

(3) $$\bar{v}=\frac{Rz_{dec}}{n}$$

where R is the B-scan frame rate. The number of frames, n , can be calculated using either the simple method, taking the first frame where the correlation falls below the threshold, or the interpolation method, in which a non-integer number of frames is calculated using the method described in Section 2.5. For both methods, Fig. 3(b) shows the average estimated speed when the stage was set to move at several constant speeds. The simple method under-estimates larger speeds quite significantly as expected. The maximum possible speed which can be predicted by the simple method is that corresponding to when a single frame ( n=1 ) is sufficient to drop below the threshold, which is approximately 2.6 mm/s with the parameters used here. In comparison, the interpolation method continues to accurately estimate speeds as high as 4 mm/s, even though the probe is under-sampling by a factor of 1.5 at this speed.

The instantaneous speed estimated as a function of time is shown in Fig. 3(c) for four velocities. The standard deviation is approximately 10% of the mean for all velocities in the range 0.25 to 4 mm/s, but there are an increased number of spurious high and low speed points for the larger speeds. Figure 3(d) shows the results of setting the motorized stage to change speed during the acquisition. Due to the way the stage was programmed, the speed briefly dropped to zero between each segment, as can be seen in the estimated speed. The processing step of masking bright pixels prior to measuring the correlation between images was found to be critical when specular reflections occurred from the tissue surface, otherwise the probe speed would be significantly under-estimated at these points, as shown in Supplement 1.

**Fig. 4.**
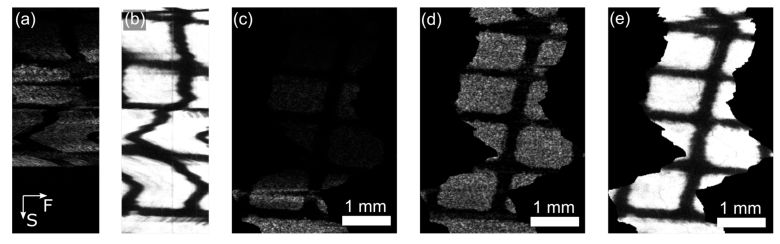
Reconstruction of OCT and fluorescence images following freehand probe scan over fluorescently-stained printed grid phantom. (a) *En-face* slice extracted from raw volume. (b) Raw fluorescence image. (c) *En-face* slice from motion-corrected volume without surface correction. (d) *En-face* slice from motion-corrected volume with surface correction. (e) Motion-corrected fluorescence image. (a)-(b) have the same horizontal scale as (c)-(e) but have no vertical scale since this depends on the instantaneous probe speed. The arrows in (a) show the direction of the fast lateral (F) and slow manual endoscope (S) scans.

The OCT probe was then moved freehand over a printed grid phantom with fluorescent highlighter applied. An example of the raw *en-face* OCT and fluorescence images are shown in Fig. 4(a,b). Severe distortion is present due to lateral motion and inconsistent out-of-plane motion, as expected. Figure 4(a) also shows the effect of axial motion of the probe; this is less apparent in Fig. 4(b) due to the lack of optical sectioning in the fluorescence channel (see Section 3.1). Figure 4(c) shows the reconstructed *en-face* image correcting only for out-of-plane and lateral motion, while Fig. 4(d) shows the result if surface-flattening is also employed. Finally, Fig. 4(e) shows the reconstructed fluorescence image. While some artifacts can be observed in the reconstructed *en-face* views, the algorithm successfully recovers the broad morphology of the sample.

## 4. Imaging results under simulated conditions

**Fig. 5.**
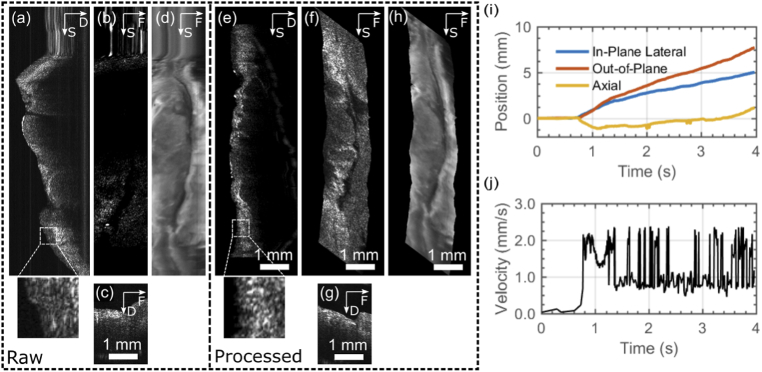
Example reconstruction from manual acquisition using endoscope for slow-axis scanning over porcine lung tissue. The raw volume is represented in (a), (b) and (c), which shows a B-scan along the slow-scan direction (a), an *en-face* slice (b) and a B-scan along the fast-scan direction (i.e. a raw B-scan) (c). The raw fluorescence data is also shown in (d). The reconstructed OCT volume is shown in (e), (f) and (g), which again shows a slow-axis B-scan (e), an *en-face* view (f) and a fast-axis B-scan (g). The reconstructed fluorescence is shown in (h). The *en-face* and fluorescence images have been rotated by 37o for display purposes. The insets show zooms on parts of the slow-axis B-scans, where the improved uniformity of the speckle pattern can clearly be seen in the processed image. Labeled arrows indicate the directions of the fast lateral (F) and slow manual lateral (S) scans and axial depth (D). The in-plane lateral, out-of-plane lateral and axial shifts detected by the algorithm are shown in (i), and the measured out-of-plane speed is shown in (j). (a) and (b) are at the same horizontal scale as the other images, but have no vertical scale since the vertical position corresponds to the time of acquisition of each B-scan. Raw data and reconstruction code is available in Ref. [44].

An example of an OCT volume and fluorescence images of porcine lung tissue (stained with acriflavine hydrochloride, as described in Section 2.), manually acquired using the endoscope for the slow-scan (as described in Section 2.3), is shown in Fig. 5. Figure 5(a-d) show raw acquisitions, while Fig. 5(e-g) show reconstructed images after processing. Figure 5(c,g) show B-scans along the fast-scan direction, while Fig. 5(a,e) show B-scans extracted from the volume along the slow-scan direction. Figure 5(b,f) show *en-face* images extracted from the volume. There was considerable in-plane lateral motion during this scan, and so the *en-face* and fluorescence images have been rotated by 37 degrees for convenient display. The effect of the algorithm in removing the motion artifacts near the top of the images can clearly be seen. The surface flattening effect can also be clearly observed by comparing the slow-axis B-scans and by observing the more constant intensity along the *en-face* image in Fig. 5(f).

Figure 5(i) shows the relative probe position as a function of time calculated by the algorithm, while Fig. 5(j) shows the calculated speed in the slow-scan (out-of-plane) direction. For most of the scan the measured probe speed in the out-of-plane direction was approximately 1 mm/s, although there are a large number of spikes up to around 2 mm/s which could be a result of jerky motion leading to sudden decorrelation.

**Fig. 6.**
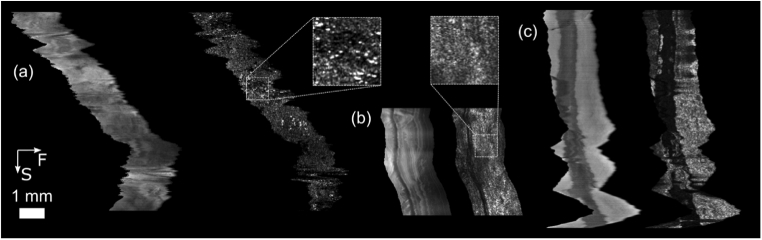
Representative pairs of fluorescence (left) and OCT images (right) from porcine *ex vivo* tissue generated when using an endoscope and manual acquisition for the slow-axis scanning. (a) Lung, (b) esophagus, and (c) trachea. The samples were stained with arcriflavine hydrochloride for 2 minutes and then rinsed prior to imaging. *En-face* slices were manually selected for display from reconstructed volumes. For display purposes, *en-face* OCT slices were contrast adjusted using the ImageJ autocontrast tool. Fluorescence images were autocontrasted to show the full dynamic range of the image. Zoomed insets show 1x1 mm regions. Arrows show direction of fast lateral (F) and slow manual endoscope (S) scans.

Further example of images manually acquired using the endoscope are shown in Fig. 6. *En-face* and fluorescence images are shown from porcine trachea in Fig. 6(a), lung in Fig. 6(b) and esophagus in Fig. 6(c). The tissue was prepared and stained as before, with the difference that, in order to simulate a typical usage scenario for this probe, we ran the probe through a plastic tube with an inner diameter of 12mm , which was placed against the opened and pinned stained tissue. All three sets of images are shown at the same scale, except for the zoom insets which show an area of 1 mm x 1 mm.

## 5. Discussion and conclusions

We have demonstrated the feasibility of using a conventional endoscope to provide the slow-axis scan for simultaneous 3D OCT and fluorescence endoscopic imaging. In experiments designed to simulate clinical imaging, slow-axis scans over ranges of up to 1 cm were performed, and *en-face* images successfully reconstructed using the speckle decorrelation and registration algorithms. While some image artifacts are often present, and the method is not robust to certain kinds of motion, the *en-face* images generally appear congruent and are much larger in the slow-scan direction than could be achieved using any miniaturized 2D endoscopic scanning mechanism. The probe was validated only using tissue labeled with a fluorescent contrast agent; further work will be required to evaluate the SNR for applications requiring imaging of intrinsic fluorescence.

Importantly, the method does not require any additional tracking systems, potentially simplifying clinical translation by allowing for more straightforward integration with existing clinical workflows. This is in comparison to other methods of tracking freehand-scanned probes such as with cameras [46], optical encoders [47] or magnetic tracking systems [48].

The reconstruction performance is primarily limited by the fast-axis scanning rate of the endoscopic OCT probe. With a B-scan frame rate of 250 Hz and a lateral resolution of approximately 20 µm, the (manual) slow-axis scan is limited to a speed of approximately 2.5 mm/s before under-sampling and hence degradation of the lateral resolution in the out-of-plane direction. The resolution determined in section 3.1 can therefore be considered a best case given that the probe was translated at a constant speed below this threshold. At speeds greater than 5 mm/s the adjacent B-scans will be almost entirely decorrelated and it is no longer possible to estimate the speed correctly. These scanning speeds are rather low and may be difficult to achieve in clinical practice. However, a relatively modest increase in the speed of the B-scan frame, for example to 1 kHz (which is technically feasible), would increase the permitted scanning speed to between 10 mm/s and 20 mm/s. The maximum speed is therefore not a limitation of the approach in general. However, sudden or jerky motion during a scan will also lead to missed areas of tissue, and even with very high fast-axis scanning speeds it is unlikely that these artifacts could be avoided entirely.

The manual slow-scan direction must be roughly perpendicular to the fast-scan direction. While some in-plane motion parallel to the fast-scan direction can be tolerated, and is corrected by the registration procedure, large amounts of in-plane motion will reduce the accuracy of the speckle decorrelation algorithm and lead to a smaller area of tissue being imaged. It would therefore not be possible to simply insert the OCT probe into the working channel of an endoscope; there would need to be some way to check that the orientation aligns with one axis of the endoscope bending motion. This could be intrinsic, through mechanical design forcing the probe to be inserted correctly, or by visualizing the fluorescence excitation on the endoscope camera view and manually rotating the probe. Alternatively, it may be desirable to permanently build the OCT probe into the endoscope, it which case the alignment would be fixed during manufacture.

The speckle decorrelation algorithm cannot detect a change in the direction of the out-of-plane motion. While in principle the endoscope operator could ensure that the endoscope is translated only along a single direction during the scan, patient motion or inadvertent endoscope motion could result in the same area of tissue being scanned over more than once. However, it may be possible to detect and remove these occurrences through image analysis, and real-time display would help the operator to identify when they have occurred.

These limitations are partially mitigated by allowing the operator to see the *en-face* image being assembled in real-time, providing visual feedback on the scanning speed and direction and allowing any errors to be more easily identified as they occur. We have demonstrated that the algorithm is computationally inexpensive and can readily be applied in real-time for a B-scan rate of 250 Hz. To allow for higher frame rates, Complex Master-Slave OCT could be used to reconstruct only a limited axial range around the known surface height.

We have therefore shown that manual slow-axis scanning in combination with correlation-based probe tracking is a promising approach for endoscopic forward-viewing OCT and fluorescence imaging. The validation studies reported here make use of simple phantoms that do not accurately simulate patient anatomy, using relatively large lumens. The true target anatomy will influence the range and stability of endoscope motion, and patient motion will also introduce an additional challenge to motion correction. The next steps in development should therefore involve more extensive studies using more realistic phantoms incorporating simulated patient motion, or animal models. To aid these further investigations, we have made the code for reconstructing OCT volumes and fluorescence images available with this report [44,45].

The approach may also have other applications, such as for lower-cost hand-held probes for external body imaging or industrial inspection and non-destructive testing. Alternatively, it may be applied to endoscopic probes designed to be operated with mechanical or robotic scanning systems (such as robotic surgical systems). Indeed, for robotic systems it should be possible to use the speed estimation from the OCT speckle decorrelation for closed-loop robot control, leading to more precise and controlled imaging.

## Data Availability

Data and Matlab code underlying the results presented in this paper are available in Code 1 [44]. The real-time LabVIEW code is also available in Code 2 [45].

## Supplemental document

See Supplement 1 for supporting content.
