# Supplementary material for: Endoscopic en-face optical coherence tomography and fluorescence imaging using correlation-based probe tracking
Source: Biomed Opt Express. 2022 Jan 14;13(2):761–76. doi: 10.1364/BOE.444170 (PMC8884237; doi:10.1364/BOE.444170)
Supplement: Supplementary file 1 [file boe-13-2-761-s001.pdf]

Supplemental Document

Biomedical Optics EXPRESS

Endoscopic *en-face* optical coherence tomography and fluorescence imaging using correlation-based probe tracking: supplement

MANUEL J. MARQUES,[1,3,*] MICHAEL R. HUGHES,[1,3] ADRIÁN F. UCEDA,[1] GRIGORY GELIKONOV,[2] ADRIAN BRADU,[1] AND ADRIAN PODOLEANU[1]

[1]*Applied Optics Group, Physics and Astronomy, Division of Natural Sciences, University of Kent, Canterbury CT2 7NH, United Kingdom*
[2]*Institute of Applied Physics RAS, Nizhny Novgorod, Russia*
[3]*Both authors contributed equally to this publication*
[*]*M.J.Marques@kent.ac.uk*

This supplement published with Optica Publishing Group on 14 January 2022 by The Authors under the terms of the Creative Commons Attribution 4.0 License in the format provided by the authors and unedited. Further distribution of this work must maintain attribution to the author(s) and the published article's title, journal citation, and DOI.

Supplement DOI: https://doi.org/10.6084/m9.figshare.17430407

Parent Article DOI: https://doi.org/10.1364/BOE.444170

Endoscopic *en face* optical coherence tomography and fluorescence imaging using correlation-based probe tracking: supplemental document

1. COMPARISON OF SURFACE DETECTION METHODS

The method to determine the out-of-plane speed requires that a region of interest (ROI) is extracted from within a scattering region of the sample. In practice this means placing the ROI just under the top surface. We compared five different approaches for determining the location of the top surface of the sample in the B-scan:

- **Method 1.** Convolution of each A-scan in the B-scan with a step function (5 pixels of -1, 5 pixels of 1). The position of the peak intensity of the result for each A-scan is then taken as the position of the surface for that A-scan.
- **Method 2.** As Method 1, but using correlation instead of convolution.
- **Method 3.** As Method 2, but using normalized cross correlation.
- **Method 4.** Binarization of the B-scan (using a threshold of twice the mean pixel value for the image) followed by morphological open and close operations with disc-shaped structuring element of 30×30 pixels (285×300 µm). The first non-zero pixel in each A-scan is then taken to be the surface position for that A-scan.
- **Method 5.** Applying a Sobel filter and then taking the position of the peak intensity in the result for each A-scan as the location of the surface position for that A-scan.

For each approach, three different pre-processing steps were also tested: an auto intensity adjustment (Matlab imadjust, using 1% saturation high and low), a Wiener filter, and a 3x3 moving average filter.

All methods were compared against manual segmentation of a test set of B-scans extracted from 10 data volumes, with three B-scans analysed per volume. The first three volumes were taken from *in-vivo* human skin imaging, while the other seven were taken from *ex-vivo* ox tripe. The RMS difference between the manual and automatic surface position was then averaged across all the datatsets. The results, grouped by method and image pre-correction, are shown in Fig. S1(a).

The Sobel filter with pre-processing using the Wiener filter (Fig. S1(a)(ii)) resulted in the lowest average RMS error across the datasets (0.13 mm) . However, while this was slightly better than the binarization and morphological operations method (0.29 mm), the binarization method is computationally much simpler. Small errors with respect to the manual segmentation are not of great importance as the ROI will still cover approximately the desired area of tissue. The binarization and morphological method (Fig. S1(a)(i)) was therefore used throughout this study.

Fig. S1(b) compares the results of these two methods on the images on which they performed best and worst: an example of *in-vivo* human skin, on the left, and *ex-vivo* tripe, on the right. In both cases, the median top surface position (and hence the location used for the top of the ROI) is the same to within a few pixels (i.e. a few 10s of µm).

2. EXAMPLES OF INTERMEDIATE PROCESSING STEPS

As an example, the processing steps for locating the approximate axial position of the tissue surface in a B-scan using the selected method are shown for *ex vivo* imaging of porcine lung tissue in Fig. S2. The raw image shown in Fig. S2(a) is thresholded and binarized, as shown in Fig. S2(b), and morphological open and close operations are performed, as shown in Fig. S2(c). The location of the first non-zero pixel in each column is shown overlaid on the B-scan in Fig. S2(d). The

median value of these surface heights, which is used to position the region of interest (ROI) for correlation measurements, is shown in Fig. S2(e).

Pre-processing steps prior to computing the correlation between two images are illustrated together with the correlation step in Fig. S3. The full B-scan shown in Fig. S3(a) has a ROI extracted, as shown in Fig. S3(b). The mean filtered version of this ROI, Fig. S3(c) is subtracted from the original ROI to obtain an image with increased speckle contrast, Fig. S3(d). This is then Gaussian filtered to reduce noise, as shown in Fig. S3(e). Thresholding, followed by a morphological open operation, produces a mask which is shown in Fig. S3(f) and overlaid as red pixels on the ROI in Fig. S3(g). Only pixels which are not masked, i.e. those which are black in Fig. S3(f), are used in the correlation calculation, to avoid saturated pixels affecting the results. The cross-correlation is taken between the masked image and a masked reference image to determine the in-plane shifts. After correction for the in-plane shifts, the correlation is calculated between the reference image and the current image (with the mask still used to remove bright pixels). If the correlation is below the threshold, the B-scan is added to the volume and the current image becomes the reference image.

3. EFFECT OF ROI POSITION

The boundaries of the region of interest (ROI) for correlation between two images are selected based on the average surface height for the first of those images. Since the surface topography will vary, it is important that the correlation and hence the estimate of the probe shift is not overly sensitive to the exact axial position of the ROI. Figure S4 shows the effect of adjusting the vertical position and size of the ROI for a dataset collected with the probe moved by a translation stage for 1 s at a speed of 2 mm/s over *ex-vivo* chicken breast tissue. The measured speed, shown in Fig. S4(a) is approximately 2 mm/s everywhere except in the lower left corner of the plot which represents ROIs which lie entirely or mainly above the surface of the tissue. The standard deviation of the measured speed over the course of the scan is similar for all combinations which do not result in ROIs largely above the surface. For results reported in the paper, a ROI height of 1 mm and offset of 0 mm was used.

4. EFFECT OF MASKING SPECULARITIES

The procedure to remove bright pixels from the correlation measurement is essential when the surface presents specular reflections. This is illustrated in Fig. S5, which shows the results of processing the same speed calibration data as used for Fig. 3 but in this case without masking of bright pixels prior to calculating correlations. From Fig. S5(a) it can be seen that the average measured out-of-plane speeds for several of the programmed stage speeds were grossly under-estimated. Figure S5(b) shows the measured speed as a function of time when the stage was moved at 3 mm/s, where it can be seen that the measured speed is highly variable (mean of 1.7 mm/s, standard deviation of 0.35 mm/s). This can be understood by inspection of the ROIs extracted from B-scans acquired at times of 0.88 s in Fig. S5(c) and at 0.48 s in Fig. S5(d). For the frame at 0.48 s, there are obvious specular reflections at the tissue surface which dominate the correlation measurement and result in a slower decorrelation with distance (and hence lower measured speed). When masking is performed, these bright pixels on the surface are not included in, and hence do not affect, the correlation calculation. Note that the two images have been independently contrast-adjusted for display purposes.

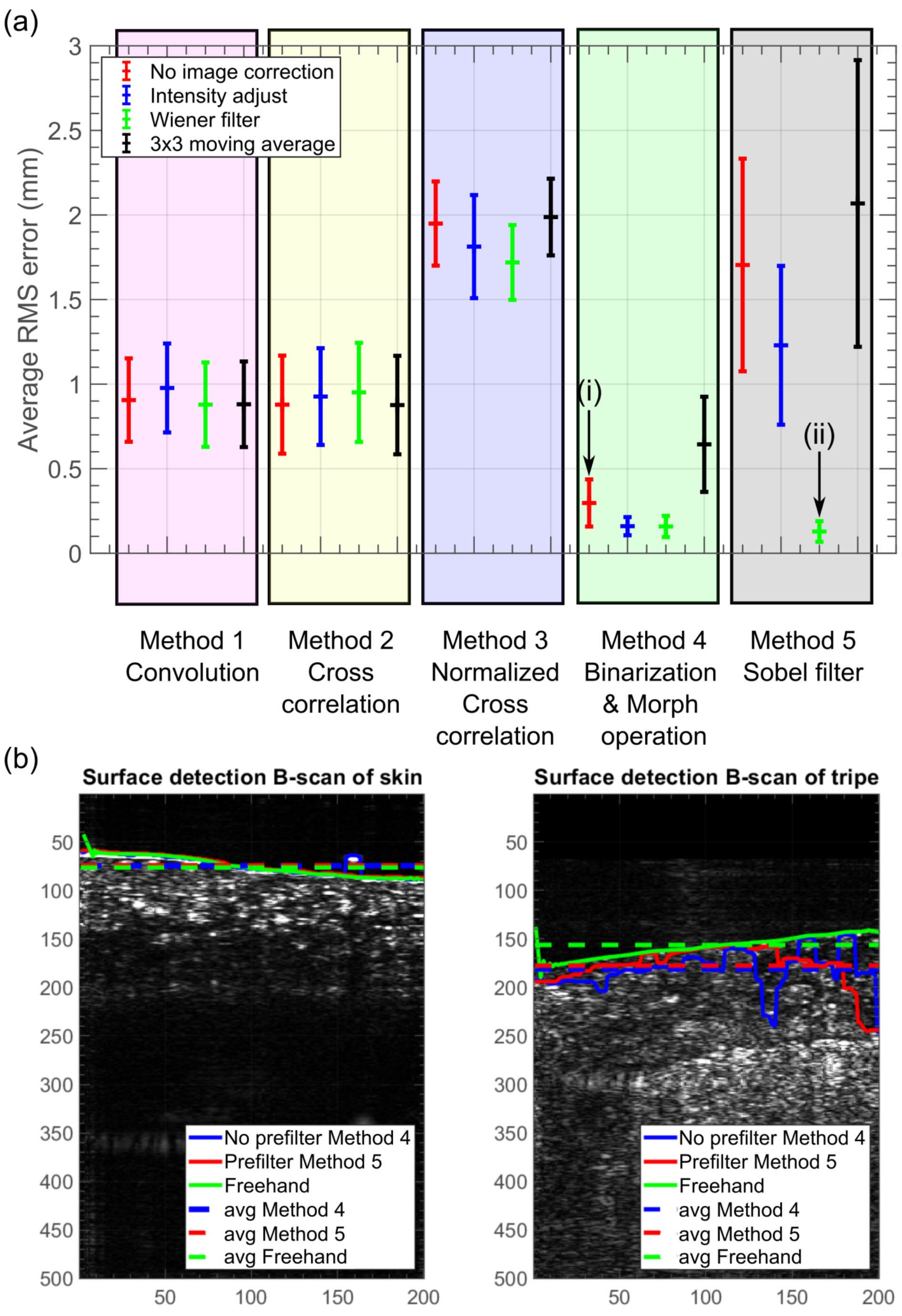

Fig. S1. (a) Average RMS error with respect to manual segmentation for each combination of surface detection approach and image pre-correction. The error bars are the standard deviation of the RMS values across 30 different test images. Arrows indicate: (i) Method 4 with no pre-correction, which was employed in the paper, and (ii) Method 5 with a Wiener pre-filtering, which provides slightly better results at the expense of more complex processing. (b) Examples of B-scans of human skin (left) and ox tripe (right). The dashed lines indicate the median position of the surface across the sample while the continuous lines show the position detected at each A-scan. The freehand registration is shown in green, the Sobel method in red, and the binarization in blue. The units are image pixels, in the vertical scale 1 pixel is 10 µm, in the horizontal scale 1 pixel is 9.5 µm.

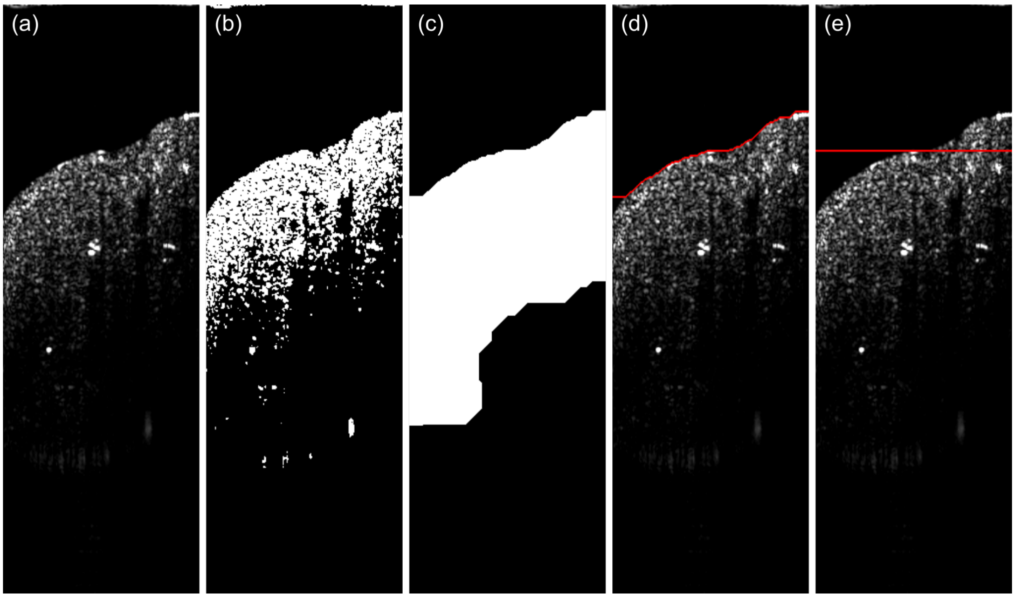

Fig. S2. Example of surface finding in a B-scan of porcine lung tissue. (a) B-scan. (b) Thresholded and binarized B-scan. (c) Morphological close and open. (d) Estimated surface overlaid on B-scan (e) Median surface overlaid on B-scan.

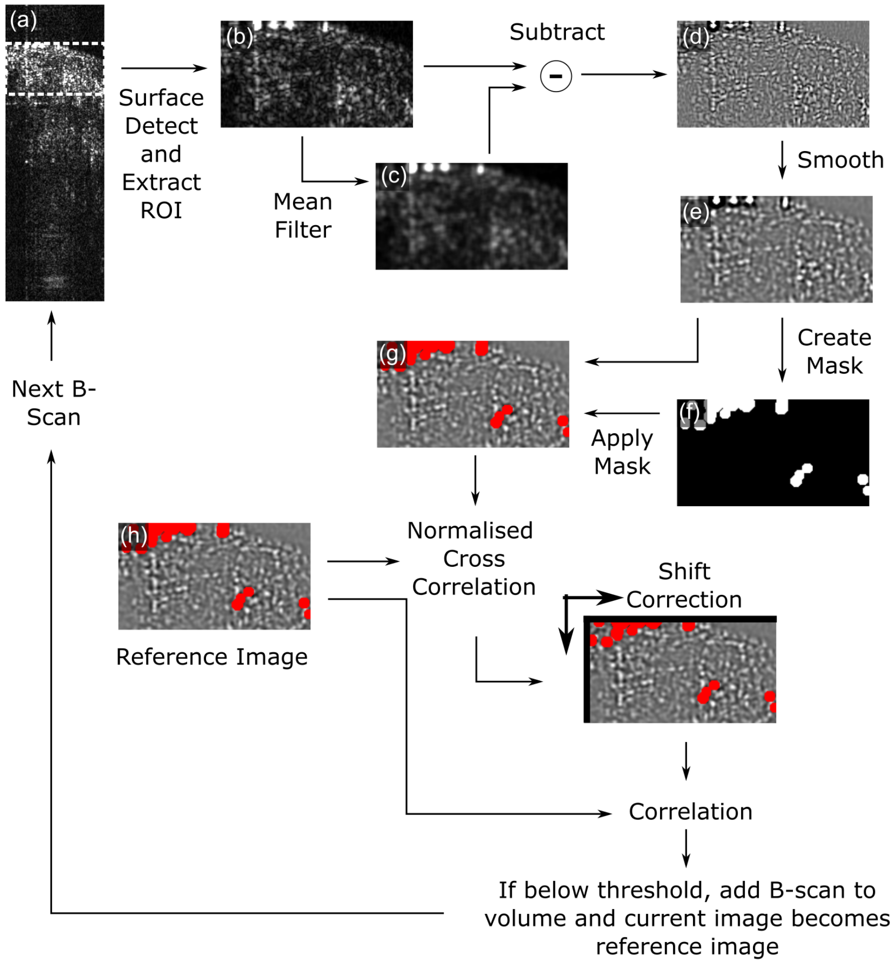

Fig. S3. Illustration of processing steps in shift calculation. (a) Complete B-scan of chicken breast tissue showing region of interest (ROI) as dashed box. (b) Extracted ROI. (c) Mean-filtered ROI. (d) Result of subtracting mean-filtered ROI from original ROI. (e) Result of a applying a 2D Gaussian filter to (d). (f) Mask calculated by thresholding and morphological open operation on (b). (g) Mask overlaid on the filtered ROI. (h) Reference image.

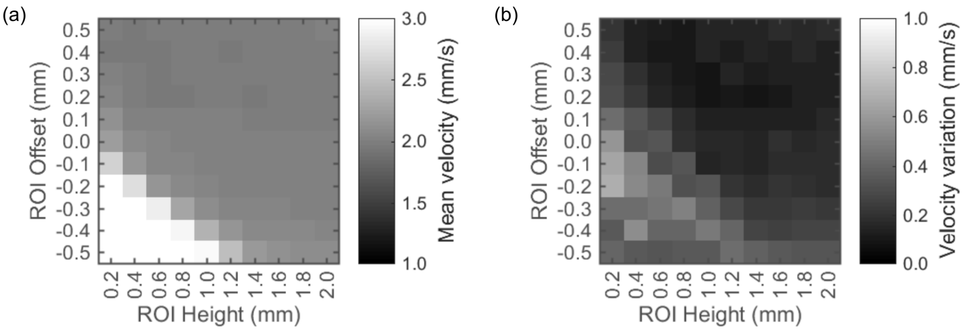

Fig. S4. Effect of vertical (i.e. axial) position (Offset) and size (Height) of region of interest (ROI) on measured out-of-plane speed. Negative offsets indicate a ROI higher in the image. The probe was translated at 2 mm/s for 1 s over chicken breast tissue using a translation stage. (a) Mean of measured speed. (b) Standard deviation of measured speed.

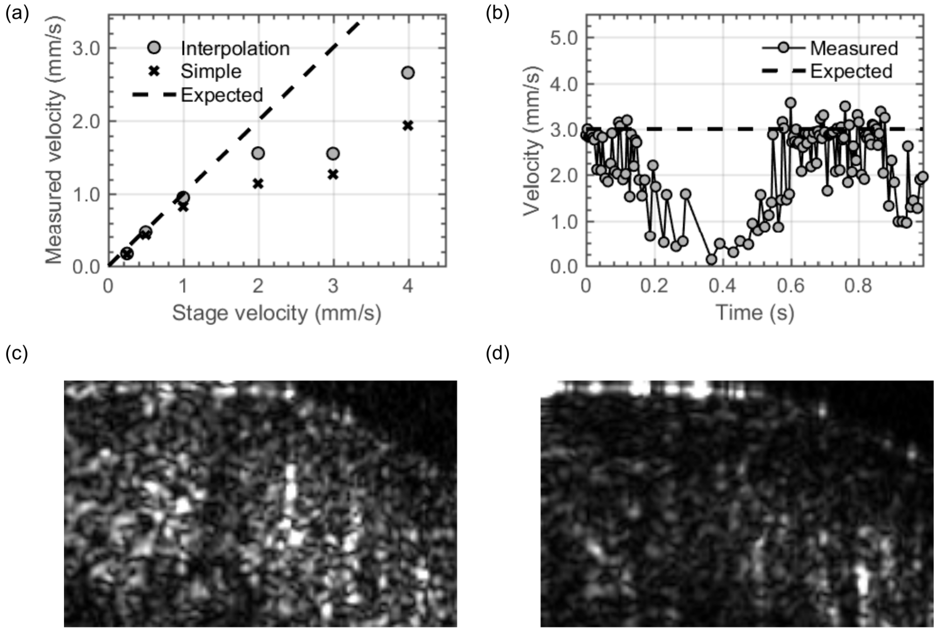

Fig. S5. Out-of-plane speed measured when masking is not used. Probe was translated at several different speeds using a motorized translation stage over chicken breast tissue. (a) Measured speeds using both simple and interpolation-based methods for six different translation stage speeds. (b) Speed measured as a function of time using the interpolation method for a translation stage speed of 3 mm/s. (c) ROI from the B-scan image acquired at time 0.88 s. (d) ROI from the B-scan image acquired at time 0.48 s. The images in (c) and (d) were both independently contrast-adjusted to show their full dynamic range.
